# Efficacy and safety of ultrasound-guided bleomycin combined with dexamethasone in the treatment of pediatric lymphangiomas

**DOI:** 10.3389/fped.2022.935470

**Published:** 2022-07-19

**Authors:** Yu-Tong Zhang, Chao Zhang, Yu Wang, Jian Chang

**Affiliations:** Department of Pediatric Oncology, The First Hospital of Jilin University, Changchun, China

**Keywords:** bleomycin, dexamethasone, lymphangiomas, pediatric, risk factors

## Abstract

**Purpose:**

This work aimed to report our experience with ultrasound-guided instillation for the treatment of lymphangiomas in children, so as to determine whether the combined use of bleomycin and dexamethasone achieved a higher response rate and a lower side effect rate.

**Methods:**

The medical records from patients with lymphangiomas between January 1st, 2013 and September 31st, 2020, were reviewed. Patients who received bleomycin combined with dexamethasone sclerotherapy were classified as the dexamethasone group, while those receiving bleomycin without dexamethasone were classified as the control group.

**Results:**

Altogether one hundred and twenty-seven patients were diagnosed with lymphangiomas. Among them, one hundred and five patients received bleomycin combined with dexamethasone injection, while the remaining twenty-two received bleomycin injection alone. The excellent rates were 89.52% [95% confidence interval (CI), 81.64–94.40%] in the dexamethasone group and 72.73% (95% CI, 52.51–92.94%) in the control group (*p* < 0.05). Additionally, the recurrence rates were 3.81% (95% CI, 1.22–10.03%) in the dexamethasone group and 13.64% (95% CI, 3.6–36.0%) in the control group (*p* > 0.05). After comparison between the two groups, the following risk factors were identified. These include >10 sacs at the initial stage of diagnosis, larger size after all injections, and response to the first injection.

**Conclusions:**

Although there was no significant difference in the recurrence rate between the two groups, this retrospective study demonstrated that the excellent response rates were dramatically improved between the two groups, suggesting that bleomycin combined with DEX was an effective and highly safe treatment for all types of pediatric lymphangiomas. Moreover, this study also identified three novel features as the significant risk factors for recurrence.

## Introduction

The efficacy and safety of diverse treatment modalities in the treatment of pediatric lymphangiomas remain controversial (1). Surgical excision has been considered by most surgeons as the gold standard of treatment for lymphatic malformations, however, it is associated with severe side effects such as high risk of recurrence (2). As such, injection of bleomycin has been investigated as an alternative therapeutic modality. Nevertheless, there are new concerns about the injection, including swelling and allergic reaction (3). Indeed, post-injection airway obstruction has been reported in children with lymphangiomas (4). Hence, several authors consider symptomatic hygroma in the neck and mediastinum as a contraindication for intralesional bleomycin injection (3, 5). According to these side effects, this work reported our experience with ultrasound-guided instillation for the treatment of lymphangiomas in children. The existing data from our center of pediatric patients were examined to determine whether the combined use of bleomycin and dexamethasone (DEX), as compared with bleomycin alone, in routine clinical practice increased the response rate and decreased the recurrence rate.

## Methods

### Data sources

This study was approved by the Ethical Committee of our hospital. Medical records from children with lymphangiomas between January 1st, 2013 and September 31st, 2020, were reviewed. In this work, all patients who were managed with bleomycin sclerotherapy with or without dexamethasone including those who underwent bleomycin sclerotherapy after surgery or as a secondary treatment, were enrolled. The following data were collected from each patient, including age, gender, site of lymphangiomas, number of injections, response to therapy, and injection complications. Our study protocols were approved by the hospital's institutional review board. All data remained anonymous.

The initial diagnosis of lymphangioma was made based on physical examination, and was subsequently confirmed by ultrasonography or magnetic resonance imaging (MRI) or both. When imaging findings and clinical features were not conclusive, surgical biopsy was planned to confirm the diagnosis (6). The tumor locations were grouped into 3 regions, including head and neck, trunk, extremities and other. According to the radiological appearance of lymphatic cavities, lymphangiomas were classified into two main types, namely, single cystic (SI) and cavernous (CA) types. Of them, the SI type was further divided into macrocystic (MA, with diameter >1 cm and cysts <5) and microcystic (MI, with diameter <1 cm and/or cysts >5) types. CA was defined as multiple cysts (with no restriction on size or number), in which solid elements contained the microscopic channels.

Patients receiving bleomycin combined with DEX sclerotherapy were classified as the DEX group, while those taking bleomycin without DEX were classified as the control group.

### Treatment

All procedures were performed under general anesthesia by the same operator (J.C.) in the operating room under the guidance of ultrasonography. Informed consent was obtained from the caretakers of patients in all instances. Notably, patients who received bleomycin without DEX were diagnosed at the early stage of this retrospective study. At first, DEX was not routinely used in combination with bleomycin until it was used to reduce an allergic reaction in one patient with tachypnea induced by huge neck lymphangioma who was also sensitive to many drugs. It was so effective that her symptoms improved gradually without any side effects, including local swelling. From then on, DEX was used in combination with bleomycin as the sclerotherapy for all patients with lymphangioma.

For patients in the DEX group, fluid in the cyst was punctured with a fine needle and aspirated as much as possible. Under ultrasonic guidance, the lymphatic fluid in the sac was aspirated by an empty needle puncture, and for polycystic patients, the lymphatic fluid was aspirated by puncture separately. Bleomycin solution (1 mg/ml normal saline solution) was injected into the cystic lesions at a dose of 0.3–0.6 mg/kg, and the maximum dose should be no more than 10 mg per injection (2, 7). In other words, fluid from the lesion was aspirated completely and bleomycin was injected at the maximum amount of 20 mL. This step was repeated to include all the major cysts, so that the drug was evenly distributed in the tumor. Patients were observed in the hospital for 2–4 h and discharged on the same day. At 2 weeks to 1 month post-injection, the patients were recalled for follow-up visits. The response was assessed clinically and radiologically, and graded as excellent response (80–100%), good response (50–80%), and no response (<50%) (4, 8). Operative complications occurring within 1 month after the primary treatment were reported, and ultrasound or MRI was repeated at 1–3 months to monitor the recurrence. The injections were repeated when the outcome was unsatisfactory.

Patients in the control group received the same procedures without the use of DEX.

### Statistical analysis

In this work, the primary outcomes were the excellent and recurrence rates. Qualitative data were presented as numbers and percentages and compared using Chi-square test. If the data were not qualified for Chi-square test, Fisher's exact test was used. Nonparametric data were compared using Mann-Whitney *U* test. Statistical significance was set at *p* < 0.05. GraphPad Prism 8.0 was employed for all statistical and image analyses. All proportions were presented as 95% CIs.

## Results

Between January 1st, 2013 and September 31st, 2019, one hundred and twenty-seven patients were diagnosed with lymphangiomas, including forty-six female and eighty-one male patients. The most common site of lymphangiomas was head and neck (n=108, 85.04%), meanwhile, trunk, and extremity involvements were noted in 11 (8.66%) and 8 (6.3%) cases, respectively. Ninety-seven patients had single cyst, including twenty-one of MA type and seventy-six of MI type, whereas the remaining thirty patients had CA type. A total of one hundred and five patients received bleomycin combined with DEX injection, whereas the remaining twenty-two underwent bleomycin injection. The characteristics of the one hundred and twenty-seven patients are summarized in Table 1.

**Table 1:** The characteristics of the one hundred and twenty-seven patients.

	**DEX group**	**Control group**
Age (mean ± se) (year)	3.34 ± 2.33	3.76 ± 3.12
Total	105	22
**Gender**		
Male	30	16
Female	5	6
**Site**		
head and neck	91	17
trunk	8	3
extremities and other	6	2
**Mainly types**		
**Single cystic**	80	17
Macrocystic	18	3
Microcystic	62	14
**Cavernous**	25	5

The median age at diagnosis of the one hundred and five patients in the DEX group was 3.34 ± 2.33 (range, 0.2–9.7) years. Ninety-one, eight, and six patients had tumors in the head and neck, trunk and extremities, respectively. In the meantime, eighteen patients were of MA type (18/105, 17.14%), sixty-two of MI type (62/105, 59.05%), and twenty-five of CA type (25/105, 23.81%). There was no difference in the response according to the tumor site. The excellent response rates of sclerotherapy in MA, MI and CA types were 94.44% (17/18), 90.3% (56/62), and 84.00% (21/25), respectively, whereas that in the whole group was 89.52%. Two injections were required in five patients (4.76%), including one of MA type, three of MI type, while the other one of CA type. Six patients required 3 or more injections, among them, 3 were of MI type and the other three were of CA type. Notably, two patients of CA type showed no response to sclerotherapy. However, from a cosmetic point of view, the appearance was greatly improved. During the follow-up, none of the patients receiving the one-time injection developed recurrence. Four patients undergoing more than two injections experienced recurrence, of them, one was of MI type while the other three were of CA type. The characteristics of patients not achieving excellent response after the first injection in the DEX group are summarized in Table 2.

**Table 2:** The characteristics of patients not achieving excellent response after the first injection in the control group.

**Group**	**Patient**	**Gender**	**Age (year)**	**Site**	**Type**	**Initial size** **(cm** × **cm)**	**Size after first injections** **(cm** × **cm) 2 weeks**	**Size after multiple injections** **(cm** × **cm)**	**Time of injections**	**Response after injections**	**Follow up**
DEX group	1	M	2.3	H	MI	6.2 × 7.3 × 4.1 A	2.0 × 3.1 × 2.9	1.2 × 1.3 × 1.3	2	Excellent	Excellent
	2	F	2	H	MA	3.8 × 6.4 × 3 A	1.5 × 2.2 × 2.2	1.1 × 1.2 × 0.9	2	Excellent	Excellent
	3	F	3	H	MI	6.8 × 5.4 × 5.2 A	3.8 × 5.1 × 3.9	1.9 × 1.7 × 1.5	3	Excellent	Excellent
	4	M	4.3	H	CA	8.7 × 7.3 × 6.5 B	5.2 × 6.4 × 4.9 (39.5%) good	2.7 × 2.5 × 2.4	3	Excellent	Recurrence
	5	M	2	H	MI	4.8 × 4.6 × 5.1 A	1.6 × 3.2 × 2.5	1.1 × 1.3 × 1.3	2	Excellent	Excellent
	6	M	1.6	E	MI	3.5 × 5.4 × 4.6 A	1.7 × 3.2 × 2.0	1.2 × 1.0 × 1.4	2	Excellent	Excellent
	7	F	1.2	H	MI	9.4 × 7.3 × 6.5 B	6.2 × 5.9 × 5.3 (43%) good	2.8 × 2.6 × 2.3	3	Excellent	Recurrence
	8	F	7.7	T	MI	19.4 × 9.1 × 15.9 A	9.2 × 7.7 × 6.3 A	1.2 × 1.3 × 1.3	4	Excellent	Excellent
	9	M	3.5	H	CA	9.4 × 4.8 × 5.7 A	5.1 × 4.0 × 3.5 (27%) good	1.8 × 1.6 × 1.7	3	Excellent	Excellent
	10	M	6.4	H	CA	6.5 × 9.7 × 5.5 A	6.3 × 7.7 × 5.5 (78%) no response	4.5 × 6.4 × 5.5	3	Good	Recurrence
	11	F	3.3	H	CA	5.7 × 8.4 × 6.8 B	5.4 × 7.0 × 6.6 (76%) no response	5.6 × 4.3 × 5.1	3	Good	Recurrence
Control group	1	M	3.3	H	MI	4.8 × 6.7 × 5.2 B	2.2 × 3.1 × 2.6	1.1 × 1.2 × 1.3	2	Excellent	Excellent
	2	F	3.2	H	MI	4.4 × 4.8 × 4.7 A	2.2 × 2.2 × 2.4	1.1 × 0.9 × 1.2	2	Excellent	Excellent
	3	F	1	H	MI	6.5 × 9.7 × 3.2 A	2.5 × 3.6 × 3.5	1.4 × 1.0 × 1.3	2	Excellent	Excellent
	4	M	3.2	H	MI	5.7 × 8.4 × 5.8 B	5.5 × 7.3 × 5.3 (76.6%) no response	3.4 × 5.0 × 4.1	2	Good	Recurrence
	5	M	5	H	MI	5.7 × 8.5 × 7.4 B	5.4 × 6.8 × 7.2 (73.3%) no response	3.0 × 4.0 × 3.3	2	Excellent	Recurrence
	6	F	6.8	T	CA	6.9 × 7.6 × 8.3 B	6.4 × 7.2 × 6.7 (71%) no response	3.5 × 4.2 × 2.5	5	Excellent	Recurrence

*M, male; F, female; DEX, dexamethasone; H, head and neck; E, extremities and other; T, trunk; CA, cavernous; MA, macrocystic; MI, microcystic; A, <10 sacs at the initial diagnosis; B, more <10 sacs at the initial diagnosis*.

Accordingly, for patients in the control group, the median age at diagnosis was 3.76±3.12 (range, 0.5–8.9) years. Seventeen patients had lymphangiomas in head and neck, three in the trunk, and two in the extremities. Moreover, three patients were of MA type (3/22, 13.63%), fourteen of MI type (14/22, 63.64%), and five of CA type (5/22, 22.73%). A total of sixteen patients achieved excellent response, including three of MA type, nine of MI type and four of CA type, and no recurrence was reported among these patients during the follow-up. The excellent response rates of sclerotherapy in the MA, MI and CA types were 100, 64.29, and 80%, respectively, while that in the whole group was 72.73%. Five patients of MI type required two injections, while one of CA type needed five injections. Three patients receiving more than two injections developed recurrence, including two of MI type and one of CA type. The characteristics of patients not achieving excellent response after the first injection in the control group are also summarized in Table 2.

Injection-associated swelling was common but mild. None of our patients had post-injection airway obstruction, even though lymphangiomas were located near the main trachea. Besides, no facial or phrenic nerve palsy or hoarseness was reported in our patients. Infections were not reported as well, since the puncture site was routinely disinfected more than three times.

The excellent response rates were 89.52% (95%CI, 81.64–94.40%) in the DEX group and 72.73% (95%CI, 52.51–92.94%) in the control group. Upon Chi-square test, there was a significant difference in the excellent response rate between the two groups (*p* < 0.05). The median follow-up was 3.3 (range, 1–7) years, and the most frequent complication was recurrence, with the recurrence rates of 3.8% (*n* = 4) and 13.64% (*n* = 3) in the control group occurring from 2 to 5 years. Moreover, the recurrence rates were 3.81% (95%CI, 1.22–10.03%) in the DEX group and 13.64% (95%CI, 3.6–36.0%) in the control group, although there was no significant difference between the two groups upon Chi-square test (*p* > 0.05).

After comparison between the two groups using Chi-square test, the identified risk factors for recurrence included more than ten sacs at the initial diagnosis, larger size after all injections, and response to the first injection (*p* < 0.05).

## Discussions

Different from hemangioma, lymphangiomas do not resolve spontaneously, therefore, it is essential to treat these lesions (9). Nowadays, bleomycin injection for the treatment of lymphangiomas has been proposed as the common therapeutic approach due to the complications of surgery (4, 10–12). Nonetheless, as suggested in some studies, sclerotherapy is not suitable for MI and CA types due to the small size of vessels and cysts (4, 6). Interestingly, at the beginning, DEX was used in combination with bleomycin to avoid allergic reaction. Along with the excellent response shown in several MI and CA patients, we confirmed the efficacy of DEX in combination with bleomycin in treating all types of lymphangiomas.

There was no significant difference in the recurrence rate between the two groups (3.81 vs. 13.64%), which might be due to the relatively small sample size in the control group. The excellent response rate was 72.73% in the control group, while that was 89.52% in the DEX group, both of which were improved significantly. DEX is frequently applied as a clinical anti-inflammatory drug, which plays a certain role in angiogenesis, as evidenced by research on the inhibition of tumor angiogenesis (13, 14). At present, the exact mechanism of DEX in the treatment of lymphangiomas remains unclear. Nevertheless, it is hypothesized that MI and CA types are less responsive to sclerotherapy (15), since the contractile lymphatic cisterns are situated more deeply in the subcutaneous tissue (16). Moreover, the small dimensions of the cysts or channels have stopped the flow of drugs. DEX exhibits a particularly strong tissue permeability, especially for the semi-permeable membrane (17, 18). Besides, it is reported in a study that, DEX at a high concentration (100 umol/L) inhibits the proliferation of human umbilical venous endothelial cells (hUVECs) and induce the apoptosis of endothelial cells (ECs) (19). Meanwhile, the precise mechanism of action of bleomycin in sclerotherapy remains unclear. Recently, it has been demonstrated that bleomycin can disrupt the tight junction of ECs, because endothelial-mesenchymal transition (EMT) is noticed after bleomycin treatment (20, 21). It is unclear whether bleomycin shows a synergistic effect with DEX. Moreover, due to the rarity of lymphangiomas and the lack of animal models, it is difficult to confirm the exact mechanism of DEX combined with bleomycin in treating lymphangiomas.

It has been reported that MA lymphangiomas allow easy aspiration of the contents and thus respond well to intralesional injection of bleomycin. On the other hand, MI and CA lymphangiomas do not show a good response to this treatment (2, 7, 22). Indeed, in the control group, MI type (64.29%) had the lowest excellent response rate, while MA (100%) had the highest excellent response rate, and CA had the moderate excellent response rate. Nonetheless, in the DEX group, all the three types achieved very high excellent response rates, with the lowest one being observed in the CA type (84%). According to our data, bleomycin combined with DEX was effective not only for MA type, but also for MI and CA types.

To our knowledge, no existing statistical analysis has verified the risk factors for treatment recurrence, due to the rarity of pediatric lymphangiomas. Our multivariate results revealed three novel features as the significant risk factors for recurrence after injection of bleomycin, namely, more than 10 sacs at the initial diagnosis, response of patients to the first injection, and the size of the capsule after multiple treatments.

Seventeen patients in two groups required more than two injections, among them, a total of seven developed recurrence. Moreover, the risk of recurrence dramatically increased in patients with more than 10 sacs at the initial diagnosis. Six patients with more than 10 sacs at the initial diagnosis experienced recurrence, while only one patient with <10 sacs relapsed (*p* < 0.05). Meanwhile, the risk of recurrence was also closely related to the response of patients to the first injection. It was observed that 80% of patients who responded to the first injection (excellent) did not experience relapse, while all of the patients who did not respond to the first injection had recurrence (*p* < 0.05). Moreover, the size of the capsule after multiple treatments was also closely related to recurrence. A larger size was associated with a higher risk of recurrence (213.02 ± 49.75 vs. 28.53 ± 26.42 mm^3^, *p* < 0.05). This work noted a special case, patient No. 8 in the DEX group. The patient had a very large initial lymphangioma size. When determining whether the initial size was a risk factor for recurrence, the results showed that a smaller size had a higher risk of recurrence due to the large offset (371.81 ± 61.93 vs. 461.70 ± 842.06 mm^3^, *p* < 0.05). This result did not make sense. However, patients with a large size at the initial diagnosis had a dramatically increased risk of recurrence, while this patient was excluded from the study (371.81 ± 61.93 vs. 151.11 ± 64.53 mm^3^, *p* < 0.05). It might be difficult to confirm the result due to the small sample size.

It has been reported that the major apparent advantage of bleomycin is the relatively minimal inflammatory reaction and edema post-injection (23). Minor swelling occurred in the majority of our patients. Post-injection airway obstruction, facial or phrenic nerve palsy or hoarseness, and infections were not observed in our patients.

The other main reason that intralesional bleomycin injection has not been used unlimitedly in our study is the concern about the association between bleomycin and pulmonary fibrosis. It is well-known that bleomycin can induce pulmonary fibrosis (23). The safety of bleomycin has been reported when it is used in a total dose lower than 450 mg. However, the risk significantly increases when the total dose exceeds 500 mg. Typically, the upper dose limit for single use is 30 mg/m^2^ (24). Besides, impaired renal function caused by cumulative dose of bleomycin has been reported as well (25). Although our single dose and cumulative dose were within the safe limits, it is not easy to decide the effect of bleomycin injections for more than five times due to its side effects. However, the size of the capsule after multiple treatments was a confirmed risk factor for recurrence from our data. It seems that the size <2 × 2 × 2 cm is a threshold for non-recurrence, nevertheless, there are no conformed statistic data due to the relatively small numbers of patients. Therefore, how to balance the frequency of injection to achieve an excellent treatment response and minimize the side effects of bleomycin needs further discussion.

Certain limitations should be noted in our study. This was a retrospective study, and the sample size (especially for the control group) was quite small. Nevertheless, there was no significant difference in the recurrence rate between the two groups, and this retrospective study showed that the excellent response was improved dramatically between the two groups. Therefore, our study demonstrates that bleomycin combined with DEX is an effective and highly safe treatment for all types of pediatric lymphangiomas. Moreover, multivariate analysis in our study identified three novel features as the significant risk factors for recurrence, including more than 10 sacs at the initial diagnosis, response of patients after the first injection, and the size of the capsule after multiple treatments. It seems that a large size at the initial diagnosis was also a risk factor for recurrence, however, due to a special patient in the DEX group, this factor was excluded. The side effect of swelling was common but very mild. None of the patients experienced infections, post-injection airway obstruction, facial or phrenic nerve palsy or hoarseness. Further work should focus on the treatment strategies, especially on balancing the frequency of injection and side effects of bleomycin.

## Data availability statement

The original contributions presented in the study are included in the article/supplementary material, further inquiries can be directed to the corresponding authors.

## Ethics statement

The studies involving human participants were reviewed and approved by Ethical Institution of the First Hospital of Jilin University. Written informed consent to participate in this study was provided by the participants' legal guardian/next of kin.

## Author contributions

Y-TZ, CZ, and JC: made substantial contributions to design of the work, drafted the manuscript, and agree to be accountable for all aspects of the work in ensuring that questions related to the accuracy or integrity of any part of the work are appropriately investigated and resolved. YW: made substantial contributions to the design of the work revised the manuscript critically and agree to be accountable for all aspects of the work. All authors have read and approved the manuscript.

## Funding

This project was supported by the Jilin provincial Department of Finance, China (Grant No. JLSWSRCZXL2021-083 to Y-TZ).

## Conflicts of interests

The authors declare that the research was conducted in the absence of any commercial or financial relationships that could be construed as a potential conflict of interest.

## Publisher's note

All claims expressed in this article are solely those of the authors and do not necessarily represent those of their affiliated organizations, or those of the publisher, the editors and the reviewers. Any product that may be evaluated in this article, or claim that may be made by its manufacturer, is not guaranteed or endorsed by the publisher.

## Abbreviations

CA: Cavernous
Cis: confidence interval
DEX: Dexamethasone
EMT: endothelial-mesenchymal transition, hUVECs, human umbilical venous endothelial cells, MA, Macrocystic, MI, Microcystic, MRI, magnetic resonance imaging.
